# How governments influence public health research: a scoping review

**DOI:** 10.1093/heapro/daaf097

**Published:** 2025-07-07

**Authors:** Helen Jane Senior, Navid Teimouri, Michael Waller, Simon Capewell, Katherine Cullerton

**Affiliations:** School of Public Health, The University of Queensland, 266 Herston Rd, Herston, Queensland 4006, Australia; School of Public Health, The University of Queensland, 266 Herston Rd, Herston, Queensland 4006, Australia; School of Public Health, The University of Queensland, 266 Herston Rd, Herston, Queensland 4006, Australia; Institute of Population Health, The University of Liverpool, Brownlow St, Liverpool L69 3GF, United Kingdom; School of Public Health, The University of Queensland, 266 Herston Rd, Herston, Queensland 4006, Australia

**Keywords:** government, governance, public health, health policy, evidence based policy, systematic review, ethics

## Abstract

Governments can become involved in academic research to assist in public health decision-making. However, when governments become involved, the research process can be influenced away from academic research practices, jeopardizing research integrity. This review aimed to improve understanding of this by (i) establishing the extent of literature about government influence on research, (ii) detailing key characteristics of influence, and (iii) identifying gaps meriting future investigation. We conducted a scoping review to identify relevant literature by searching five electronic databases and grey literature. Two reviewers independently screened titles, abstracts, and full-text. Extracted data included the source, characteristics of the research projects, and the influence reported. Results were categorized and analysed using numerical summaries and narrative synthesis. The literature search yielded 6890 documents, with 71 eligible for full-text review. Seventeen documents met the inclusion criteria. Published between 2007 and 2021, most came from the UK (*n* = 8) and/or Australia (*n* = 11), with two coming from both. 126 modes of influence were reported, which could take multiple forms within one document and occur at any stage of the research process. The modes of influence were categorized as ‘Direct’ in 11 documents, ‘Indirect’ in 14, and/or ‘Subtle’ in 4. Influence was predominantly negative in 13 documents, with one reporting solely positive influences. This review summarizes reported instances of governments influencing the public health research process. The results highlight a need for deeper understanding of government-academic interactions and more transparent mechanisms for good practice. By fostering positive interactions, we can support beneficial population health outcomes. The protocol was registered on the Open Science Framework on 20 Aug 2023 (https://doi.org/10.17605/OSF.IO/YB7FE).

Contributions to Health Promotion

- Government policy choices are an essential component of public health services.
- By funding public health research, governments can gain valuable information and evidence to inform health-related public policy.
- However, little is known about whether government involvement in public health research is creating positive outcomes overall and, if not, what might be influencing this.
- Our scoping review aimed to explore how governments influence public health research to expand our knowledge about government influence on research and facilitate future guidance on how governments can best interact with the research they fund for beneficial outcomes, including health promotion choices.

## INTRODUCTION

Governments and government-associated entities, from now on referred to as ‘governments’, are increasingly becoming involved in public health research, often to gain evidence to assist in policy decision-making, improve understanding of population health, or examine the effectiveness of a policy or programme (OECD 2018b, World Health Organization 2022). Government involvement can be established through various mechanisms involving patronage or finance. These mechanisms include independent grants awarded to named researchers, funding awarded to a specific project, or commissioned funding typically involving a set topic or research question(s) and a pre-specified research approach.

Concern has been raised within the public health community that when external entities, such as governments, are involved in research, it might negatively impact the research process and thus compromise the resultant evidence (Holman 2008, Daube 2018, Storeng *et al.* 2019). For instance, when engaged with public health research, governments might act with political motivations and use research findings to support their position on a specific public health issue or policy (Cairney and Oliver 2017, Furtado *et al.* 2018). Additionally, it has been suggested that government influence might also be linked to particular funding arrangements, such as commissioned research, where the funder holds interests that might motivate them to influence the research away from best practice and academic research integrity (Ham 1999, Richter and Hostettler 2015, Vaganay 2016, Norwegian National Ethics Research Committees 2021). A key challenge for such government-academic public health research is, therefore, to achieve the government’s requirements for actionable and relevant evidence whilst maintaining research integrity and preserving a robust and credible evidence base to facilitate positive population health outcomes (Barnish *et al*. 2018, D’Este *et al.* 2018, Hill *et al.* 2022).

Previous studies examining the influence of external entities on health research have focused on the commercial sector, where vested interests are more obvious; a commercial entity might commission (and influence) research for financial gain rather than for improved health outcomes (Institute of Medicine (US) Committee on Conflict of Interest in Medical Research, Education, and Practice 2009, Stamatakis *et al*. 2013, Fabbri *et al*. 2018a, 2018b). Moreover, it is widely recognized that when commercial entities commission research, this can produce a distorted evidence base, with potentially detrimental effects on population health (Fabbri *et al*. 2018a, 2018b, Greenhalgh 2019, Maani *et al.* 2020). Such influence can occur at any stage of the research process, from setting the research agenda (Testoni *et al.* 2021) to the framing of evidence (Al-Rawi 2023). For example, a food company may directly fund researchers to conduct research that supports the use of specific commercial products or services (Ahern *et al.* 2011, Gornall 2015). Equally, a food company may indirectly influence research by commissioning public health projects on topics that deflect attention away from their products that may be associated with adverse health outcomes or that seek to cast doubt on evidence about their products’ harms (Leslie 2016).

Importantly, when influence occurs, this may place academic research practices at risk of manipulation, resulting in less rigorous academic standards (Kaiser 2003, Teixeira *et al*. 2014, Edwards and Roy 2017) and the generation of evidence that is ‘favourable’ to the funder (Lesser *et al.* 2007, Bes-Rastrollo *et al.* 2013). For example, the tobacco industry's involvement in public health research has been well documented, revealing how external entities can systematically manipulate public health research and evidence to achieve their desired outcomes (Barnes and Bero 1998, Bero 2005, Ulucanlar *et al*. 2016, World Health Organization 2019, Hird *et al.* 2022). Similarly, governments may direct research towards creating an evidence base that justifies their policies or public health decisions rather than creating evidence that best reflects the population’s health needs (Bozeman and Sarewitz 2011, Jessani *et al.* 2018, Blagden 2019, Marks 2019). Therefore, if governments can influence public health research, this can not only impact the quality of public health evidence but also distort subsequent evidence utilization, decision-making, and policy choices (Cairney and Oliver 2017, Furtado *et al.* 2018), with potentially significant implications for population health (Fabbri *et al*. 2018a, 2018b, McKee and Stuckler 2018, Maani *et al.* 2020).

Despite the potential for governments to influence public health research, and considering the significant changes that are occurring in public health as a consequence of the Trump presidency, there seems to be a striking absence of literature regarding governments’ influence on the research process. This scoping review, therefore, aimed to systematically examine what is known from the literature about whether governments influence the public health research processes and, if so, how government influence might manifest. We achieved this by identifying the extent of the literature regarding government influence on public health research, describing and categorizing the characteristics and modes of government influence, and how this might positively or negatively impact the research direction or process, including any association with the type of funding arrangement. This review focused on government-funded research, where the government directly pays an academic institution to conduct research on its behalf, in contrast to research carried out within government-run or overseen research centers. This choice responds to a practical need to constrain the review to a realistic size and our ability to obtain documents relating to this topic.

## METHODS

We conducted a scoping review about government influence on the public health research process to identify the available literature globally, highlight knowledge gaps, and examine what has been documented about this topic (Peters *et al.* 2017, Munn *et al.* 2022). A scoping review was chosen as it accommodates the diverse and heterogeneous literature relating to government influence (Grant and Booth 2009, Peters *et al.* 2015). We included grey literature to capture broader literature from non-peer-reviewed sources, as we were concerned that limiting the search to only peer-reviewed documents would result in few included studies and may omit relevant policy-related literature. Our review was informed by the JBI guidance for conducting scoping reviews (Peters *et al.* 2020). The review protocol was registered on the Open Science Framework on 20 Aug 2023 (https://doi.org/10.17605/OSF.IO/YB7FE).

### Search strategy

Searches were developed with the assistance of medical librarians to identify relevant primary peer-reviewed literature, plus grey literature, which included published and unpublished documents from non-peer-reviewed sources. We followed the JBI ‘Population, Concept, and Context’ Framework (Peters *et al.* 2017) (Supplementary Material S1).

The eligibility criteria (Supplementary Material S2) were developed to capture the extent of the literature regarding modes of influence from governments relating specifically to public health research. The criteria were established following initial exploration of the literature, consultation between the research group members, and informed by the literature reviewing commercial sector influence on public health research (Smith *et al*. 2013, Savell *et al*. 2014, Fabbri *et al*. 2018a, 2018b). The key search terms were derived from the concepts of public health, academic research, government, and influence. Additionally, keywords and terms were harvested from relevant articles identified in preliminary literature searches via ‘pearl-growing’ (Booth *et al*. 2016, p. 115).

### Literature searching

Literature searches were conducted in August 2023 and repeated in January 2025. The searches included five electronic databases: Embase, PubMed, Web of Science, Scopus, and Google Scholar. Additionally, citation alerts were set to run until January 2025, utilizing ‘pearl’ articles to ensure we captured the most current literature. We searched for terms within the title, abstract, and keywords, limited to English language and Human (Supplementary Material S3). All identified document citations were downloaded into EndNote 20 (Clarivate 2023) and uploaded into Covidence (Covidence 2023), where duplicates were removed. We searched the grey literature via Google Chrome’s advanced search functions. This search involved using combinations of search terms and was limited to ‘file type: pdf’ and ‘language: English’. As suggested by Godin *et al.* (2015), the first 100 items of each search were screened using the title and text section provided. All documents identified for inclusion via Google were ‘bookmarked’ on the browser toolbar within a labelled folder and individually ‘screen-grabbed’ with a time and date to maintain traceability. The documents were then manually entered into EndNote and transferred to a file in Covidence for screening. Additional articles suggested by a topic expert (K.C.) were screened for inclusion.

### Document selection

Titles and abstracts/summaries were screened against the inclusion criteria for eligibility by H.J.S., with 10% double-screened by N.T. The abstract, executive summaries, or table of contents (whichever was available) were screened for grey literature sources. Disagreements were resolved by discussion between H.J.S. and N.T. All relevant documents underwent full-text review against the inclusion criteria by H.J.S., of which N.T. double-screened 10%. Where there was uncertainty, the third reviewer (K.C.) assessed the document’s eligibility, and disagreements were resolved. Additionally, all included documents underwent backwards searching using the reference lists and forward searching on PubMed (using ‘similar articles’ and ‘cited by’ functions) and Google Scholar (using ‘related articles’ and ‘cited by’ functions). The articles identified using the ‘pearl-growing’ technique (Booth *et al*. 2016) were added to the screening process. The search and selection process was documented as a Preferred Reporting Items for Systematic Reviews and Meta-analyses extension for the scoping reviewflow diagram (Tricco *et al.* 2018).

### Data charting and synthesis

We extracted data from documents meeting the inclusion criteria using a data collection tool developed by H.J.S. (Moon and Rao 2021). As outlined in Arksey and O’Malley’s (2005) methods for scoping review, the tool was piloted on the first three studies to ensure accuracy (Arksey and O’Malley 2005). H.J.S. extracted the data using Covidence software. Information was extracted relating to the nature of the source, the research project(s) affected by government influence, and the mode of influence documented. No critical appraisal was performed following standard practice for scoping reviews (Peters *et al.* 2017). We categorized the extracted data using content analysis (Bengtsson 2016). Informed by Vie’s qualitative study investigating modes of influence that occur in scientific commissioned research (Vie 2022), we categorized the modes of influence as ‘Direct’, ‘Indirect’, or ‘Subtle’. Vie’s framework conceptualizes influence in funded research as: ‘direct influence’ being an explicit action(s) applied to the research process itself, ‘indirect influence’ being an action(s) applied by someone or something that will secondarily impact the research process, and ‘subtle influence’ refers to influence that is not perceived or noticed but will change the course of the research cycle in a more nuanced way. We further categorized the timing of influence using the established concept of the research cycle: inception, conduct, and dissemination (Forskningsetikk 2022). H.S. conducted the initial identification of modes of government influence across all included documents. In total, 126 different modes of influence were identified, which were then mapped into the broader categories. The grouped findings were analysed using descriptive numerical summaries and narrative synthesis, and the results were presented in relation to the review objectives.

## RESULTS

### The included literature

**Figure 1.**
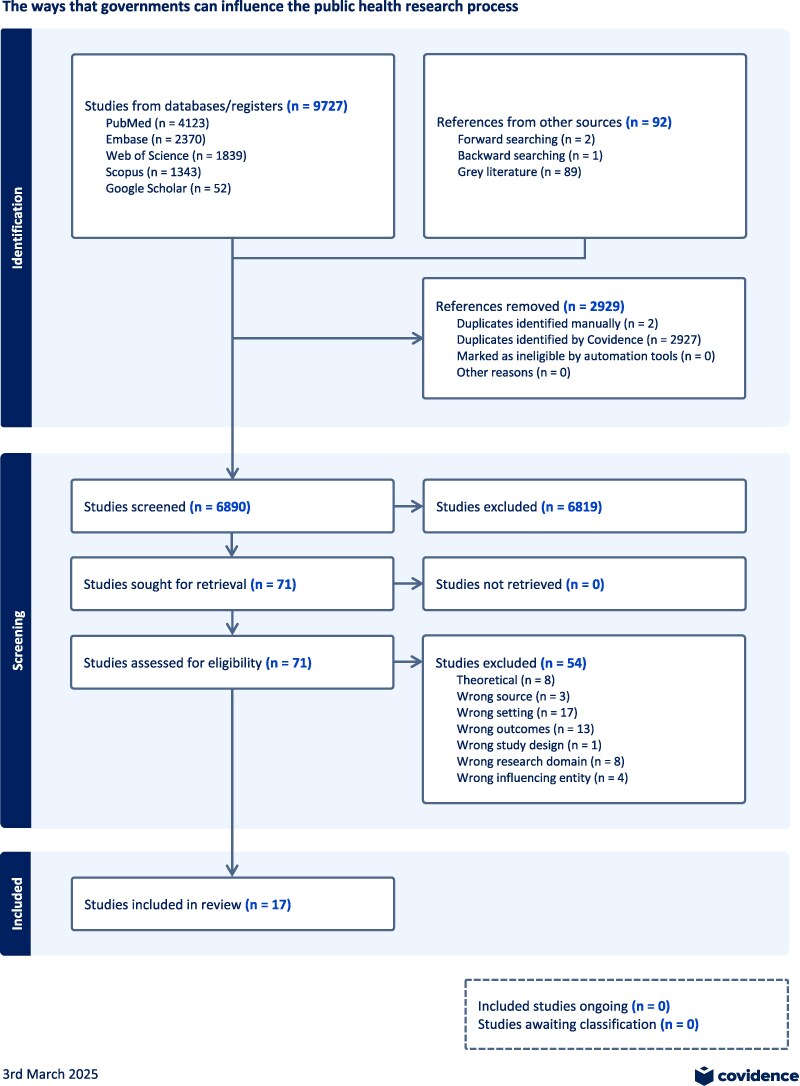
PRISMA flow diagram.

Our database and grey literature searches yielded 6890 unique sources, of which 71 were eligible for full review, including three identified following forward and backward citation searches and 89 from the grey literature. In total, we identified 17 documents describing government influence in the public health research process (Fig. 1).

Seventeen relevant documents (Yazahmeidi and Holman 2007, Smith 2010, 2014, Haynes *et al.* 2011, Gornall 2014, Katikireddi *et al.* 2014, Kypri 2015, Sedley 2016, Miller *et al.* 2017, Gordon *et al.* 2018, Ries and Kypri 2018, Storeng and Palmer 2019, Williamson *et al.* 2019, McCrabb *et al.* 2021, Newson *et al.* 2021, Warin and Moore 2021) describing government influence in the public health research process were identified (Table 1). Published between 2007 and 2021, most came from the UK (*n* = 8) and/or Australia (*n* = 11), with two documents coming from both countries. The predominant focus of the documents related to research that created evidence that informed, supported, or evaluated policy, with seven specifically reporting how the government influenced evidence for policy-making. Of those which were not directly related to policy, four reported instances where the government had blocked publications, two were about government-academic contracts, and five focused on related topics, such as research integrity or relational interactions. The majority regarded government influence from a negative (*n* = 8) or neutral perspective (*n* = 8), with only one source taking a positive view of government influence. The data within the 17 documents came from various sources, including documents, funding contracts, and individuals (including researchers and government) reporting their experiences. Furthermore, the data pertained to different contexts, such as specific public health projects, governmental departments, health policy agencies, and the academic environment. Most of the documents came from scholarly peer-reviewed journals (*n* = 15), 13 of which were academic research articles, and 2 were viewpoint/opinion articles. The grey literature search yielded two additional documents: a report and a media article. From the documents involving academic studies, a broad range of research approaches was described. Four studies used surveys or questionnaires, seven used interviews, six used document/literature analysis, and three reported case studies. Over half used qualitative approaches (*n* = 10), five used quantitative methods, and one used mixed-methods.

**Table 1.** Characteristics of included documents.

Citation	Year of publication	Author(s)	Title	Type of source	Country(ies) of publication	Study design	Methodology used
The LSE GV314 Group (2014)	2014	The LSE GV314 Group	Evaluation under contract: government pressure and the production of policy research	Peer-reviewed journal article (study)	UK	Survey	Quantitative
Gordon *et al*. (2018)	2018	Gordon *et al*.	A priority-driven, policy-relevant research programme to support a response to blood-borne viruses and sexually transmissible infections in NSW, Australia	Peer-reviewed journal article (study)	Australia	Not applicable	Quantitative
Gornall (2014)	2014	Gornall	Under the influence	News/media source	UK	Interviews; document analysis	Not applicable
Haynes *et al*. (2011)	2011	Haynes *et al*.	Galvanizers, guides, champions, and shields: the many ways that policymakers use public health researchers	Peer-reviewed journal article (study)	Australia	Interviews	Qualitative
Katikireddi *et al*. (2014)	2014	Katikireddi *et al*.	Understanding the development of minimum unit pricing of alcohol in Scotland: A qualitative study of the policy process	Peer-reviewed journal article (study)	Australia; UK	Interviews; document analysis	Qualitative
Kypri (2015)	2015	Kypri	Suppression clauses in university health research: case study of an Australian government contract negotiation	Viewpoint/opinion paper (peer-reviewed)	Australia	Case study	Qualitative
McCrabb *et al*. (2021)	2021	McCrabb *et al*.	‘He who pays the piper calls the tune’: Researcher experiences of funder suppression of health behaviour intervention trial findings	Peer-reviewed journal article (study)	Australia	Survey; interviews	Quantitative
Miller *et al*. (2017)	2017	Miller *et al*.	Funder interference in addiction research: An international survey of authors	Peer-reviewed journal article (study)	Australia; UK	Survey	Quantitative
Newson *et al*. (2021)	2021	Newson *et al*.	The how and why of producing policy-relevant research: perspectives of Australian childhood obesity prevention researchers and policymakers	Peer-reviewed journal article (study)	Australia	Literature search; interviews	Mixed-methods
Ries and Kypri (2018)	2018	Ries and Kypri	Government-funded health research contracts in Australia: a critical assessment of transparency	Peer-reviewed journal article (study)	Australia	Document analysis	Qualitative
Sedley (2016)	2016	Sedley	Missing evidence: an inquiry into delayed publication of government-commissioned research	Report	UK	Case study	Qualitative
Smith (2014)	2014	Smith	The politics of ideas: The complex interplay of health inequalities research and policy	Peer-reviewed journal article (study)	UK	Document analysis; interviews	Qualitative
Smith (2010)	2010	Smith	Research, policy and funding—academic treadmills and the squeeze on intellectual spaces	Peer-reviewed journal article (study)	UK	Document analysis; interviews	Qualitative
Storeng and Palmer (2019)	2019	Storeng and Palmer	When ethics and politics collide in donor-funded global health research	Viewpoint/opinion paper (peer-reviewed)	Norway; UK	Not applicable	Qualitative
Warin and Moore (2021)	2021	Warin and Moore	Epistemic conflicts and Achilles’ heels: constraints of a university and public sector partnership to research obesity in Australia	Peer-reviewed journal article (study)	Australia	Case study	Qualitative
Williamson *et al*. (2019)	2019	Williamson *et al*.	How are evidence generation partnerships between researchers and policy-makers enacted in practice? A qualitative interview study	Peer-reviewed journal article (study)	Australia; Lebanon	Interviews	Qualitative
Yazahmeidi and Holman (2007)	2007	Yazahmeidi and Holman	A survey of suppression of public health information by Australian governments	Peer-reviewed journal article (study)	Australia	Survey	Quantitative

### The public health research projects where government influence occurred

Fourteen of the 17 included documents reported government influence in research projects relating to High-Income Countries (HICs); two included HICs and Low- and Middle-Income Countries (LMICs), and one reported influencing events solely in LMICs. The documents where government influence was reported (Supplementary Material S4) were mainly from research projects at national (*n* = 6) and regional levels (*n* = 5), with one being international in remit. Additionally, five of the documents described a combination of national and regional projects. However, no documents reported projects that were influenced by local government. Fifteen documents specified the government representative(s) involved in the research. For instance, policymakers, agencies, members of parliament, funders, and government-employed researchers were described as being involved. Additionally, private entities (*n* = 4), NGOs (*n* = 5) and practitioners (*n* = 2) were described in the documents involving collaborative projects. Regarding funding, only four documents specified that the research project(s) received government-commissioned funding, all being UK-based projects. Six further documents reported funding via other government arrangements, such as research council funding, and seven did not specify the funding source. A range of public health topics was reported as being influenced by governments. Notably, most of the reported influence involved research related to unhealthy commodities with commercial interests, such as alcohol, tobacco, and ultra-processed food. The reported government influence occurred across a diverse range of public health topics. Most cases of influence (*n* = 9) were related to applied research in the domain of implementation, monitoring, and evaluation.

### The ways government influence manifested

The 17 included documents reported 126 modes of influence in total. These modes of influence were categorized as ‘Direct’, ‘Indirect’, and ‘Subtle’ and then summarized (Table 2). Following categorization, 11 documents reported direct government influence, 14 reported indirect, and four reported subtle, with multiple categories identified within some of the documents.

**Table 2.** Summary of the modes of influence.

Mode of influence	Nature of specific influence reported^a^	Citations
**Direct**	Preventing or delaying dissemination of findings Changing information availability Prioritizing most desirable work Displaying preferences for output(s) above scientific rigour Framing of findings Changing staff involved in the project Diverting focus away from the project Controlling access to data, resources, funding, contracts, etc.	Yazahmeidi and Holman (2007), Gornall (2014), Katikireddi *et al*. (2014), The LSE GV314 Group (2014), Sedley (2016), Miller *et al*. (2017), Gordon *et al*. (2018), Ries and Kypri (2018), Williamson *et al*. (2019), McCrabb *et al*. (2021), Warin and Moore (2021)
**Indirect**	Using bribery Threatening job/contract loss Promoting the concept of research impact Creating fear of reporting undesired findings Encouraging research to reach wider policy audiences Encouraging the use of specific methodologies, approaches, language, dissemination channels, etc. Using IP as a tool to influence others Using the credibility of researchers as a divisive tool Creating relationships with the researchers to allow more influence on the research	Yazahmeidi and Holman (2007), Smith (2010, 2014), Haynes *et al*. (2011), Gornall (2014), The LSE GV314 Group (2014), Kypri (2015), Miller *et al*. (2017), Gordon *et al*. (2018), Ries and Kypri (2018), Storeng and Palmer (2019), Williamson *et al*. (2019), Newson *et al*. (2021), Warin and Moore (2021)
**Subtle**	Promoting societal impact through idealism Creating an environment where government ideology is shared Creating job/career insecurity Promoting a research environment where fear is present	Haynes *et al*. (2011), Smith (2010, 2014), Newson *et al*. (2021)

^a^Based on the research process definition, the modes of influence have been summarized into broader categories related to their point in the research cycle using descriptive analysis to condense the modes into categories.

Government influence was identified throughout all the stages of the research process (inception, conduct, and dissemination), with some modes of influence affecting the entire process. The influence was predominantly considered negative (*n* = 13). Out of the 17 documents, only 1 reported solely positive influence, with all the other positive reports being coupled with either neutral, negative, or both. To illustrate how the different forms of influence can manifest in the research process, we have provided case examples from the included documents (Supplementary Material S5)

### The motivations and consequences of government influence

The most common motivator for government involvement in the research was reported as ‘policy-related’ (*n* = 8), where the research project was designed to facilitate public health policy decisions. Other reasons stated were ‘to gain evidence’ (*n* = 5), ‘to impart control over the research’ (*n* = 4), or ‘service or programme’-related (*n* = 3) reasons. Three documents reported motivations unrelated to government activities, and five did not disclose why the government was involved in the research. The stated objectives of the research were described as primarily related to obtaining evidence to facilitate decisions involving policy or practice.

Numerous consequences of government influence on outcomes were identified (Table 3). These consequences were categorized as negative (*n* = 13), neutral (*n* = 6), or positive (*n* = 5), with some documents containing a combination. For example, negative consequences included diverting the research agenda towards specific topics, modifying the evidence base, modifying research outputs in favour of government desires, preventing research findings from being made available to the broader public, stopping a policy from being passed, or altering the decisions made relating to the research. Positive consequences were reported as being able to improve the likelihood of getting the research into policy and practice.

**Table 3.** Consequences of government influence.

Nature of the consequence(s)	Citation	Topic of research	Reported reason for government involvement	Reported consequences
**POSITIVE IMPACTS**	(Gordon *et al*. 2018)	Blood-borne viruses and sexually transmissible infections	To support the delivery of policy-relevant research, strategic advice, capacity building and communications	The influence improved the likelihood that the research generated would be used in policy and practice, created responsiveness to emerging policy-relevant research priorities, generated cost efficiencies, and streamlined management and reporting
	(Williamson *et al*. 2019)	Health and health-related	To access additional skills, gain advice, access networks, create evidence, and support evidence-based decisions	Government involvement created nuanced and relevant outputs, allowed research to align with ‘real world’ priorities, allowed long-term, trusting relationships to develop, and gained mutual benefits for all entities
**NEGATIVE IMPACTS**	(Storeng and Palmer 2019)	Sexual Health	To mitigate ‘risks’ that the outputs might pose	Tensions between researchers and the government-associated body led to investigations by the university’s Research Governance and Integrity Office, which prevented scheduled conference attendance, halted publications; the NGOs (directed by the government) controlled which findings were made public; censored the research, prevented substantiation of analysis; left the decision-making to the ethics committee; created hesitation in publishing findings that do not support the programme resulting in unpublished findings; the research is not available for future learning
	(Gornall 2014)	Alcohol	To obtain (and manipulate) evidence on the use of the minimum unit price of alcohol to reduce alcohol use and health harms	The influence may have contributed to the retraction of the decision to use minimum unit pricing for a new alcohol policy
**NEUTRAL IMPACTS**	(The LSE GV314 Group 2014)	Health policy and programmes	To evaluate specific government policy or programme activities	The modes of influence used produced the research outcomes that the government desired whilst also preserving research integrity
	(Smith 2014)	Health inequalities	To obtain evidence to use in policymaking	Government involvement led to research that was more aligned with policy needs and, therefore, more likely to have a policy impact. It guided research towards downstream determinants of health and generated evidence that resulted in less challenging policy ideas

## DISCUSSION

Our scoping review confirms that government influence occurs in public health research, yet its investigation and reporting have been limited to date. In total, we identified 17 documents reporting instances where governments influenced or attempted to influence the public health research process. Government influence could manifest directly, indirectly, or subtly and occur at any point throughout the research process. Despite the possibility of having a positive or neutral impact on the academic research process and subsequent decision-making, the majority of documents reported government influence as negative. Furthermore, our findings leave unanswered questions regarding the extent of government influence globally and whether any associated factors might increase the likelihood of government influence occurring. Importantly, we recognize that government influence will increasingly affect public health research as funding from governments via mechanisms such as government commissioning becomes more prevalent (Aagaard *et al.* 2021).

### Government influence is a public health issue

The small number of relevant documents identified by this review starkly contrasts with the larger quantity of literature reporting influence from the commercial sector and, in particular, the tobacco, alcohol and ultra-processed food industries. The ‘Science for Profit Model’ (Legg *et al*. 2021) exemplifies this disparity by demonstrating that similar strategies of influence occur across different commercial sectors. To create this model, the authors were able to synthesize the extensive body of literature on the potential for private entities to influence public health research. This significant difference in research attention might partly reflect the ‘siloing’ effect described by Maani *et al*. (2020), who highlight the need for cross-sectoral work within public health in an attempt to prevent the over-representation of research relating to specific entities, domains, or topics in the literature (Maani *et al.* 2020). Additionally, this difference in attention may be compounded by the perception that research involving governments is beneficial and of high regard (Mikhael and Norman 2021). Consequently, research about government involvement and the potential for undue influence from the government may have been overlooked.

Despite seeking to capture the global literature and in a similar way to other studies on this topic (Miller *et al.* 2017, McCrabb *et al.* 2021), the majority of documents came from the UK and Australia. This finding may reflect the various research environments that exist globally, where public health research can be funded and conducted in different ways depending on the country and resource setting (Teixeira *et al*. 2014, OECD 2018b, Aagaard *et al.* 2021). Specifically, government-commissioned funding has been more prominent in the UK and Australia, where academic public health research evidence is used in political debates (Cairney and Oliver 2017, Kenny *et al.* 2017). For example, during the recent alcohol policy debates in England and Scotland, the Sheffield Alcohol Policy Model (Lee 2020) created by researchers within the University of Sheffield, was used to facilitate the policy choices regarding the use of a minimum unit price of alcohol (Boseley 2013). Notably, this study does not aim to provide a representative description of government influence. Rather, it maps the existing research to date and identifies notable gaps in global literature.

### Governments can influence research in many ways

Multiple and diverse modes of influence were reported in the 17 included documents. These findings echo the commercial sector literature. For instance, the ‘Policy Dystopia Model’ (Ulucanlar *et al*. 2016) summarizes the diverse influencing strategies the tobacco industry has used to influence and obtain their desired outcomes. By categorizing our results using Vie’s description of influence in commissioned research (Vie 2022), we found that ‘Direct’, ‘Indirect’, and ‘Subtle’ modes of influence can all occur in public health research. Notably, subtle influence can occur throughout the research process, suggesting a potentially unrecognized or unintended way for influence to manifest. Likewise, Haynes *et al.* (2011) also flagged the more subtle approaches that governments may use to influence public health research. Here, the authors suggest that when policymakers interact with researchers, the researchers may, over time, unconsciously modify their behaviours. Specifically, researchers may create project proposals and engage in projects considered more desirable by the policymakers, thus shifting the available evidence towards ‘policy-relevant’ evidence rather than research that focuses on a population’s needs.

We found that government influence can occur at any point in the research process, with many of the documents focusing on a single stage, typically the dissemination of findings. However, our review indicates that a broad view of government influence is required, as influence can be present throughout the entire research process. This point is reinforced by Smith *et al*. in their research about the influence of the tobacco industry (Smith *et al*. 2013). They likewise observed that influence on research can be nuanced and exists throughout the research process.

### Government influence impacts the evidence base

Our findings identified examples of positive and neutral modes of government influence. However, most documents reported negative influence, whereby the government steered the research process in ways considered potentially detrimental to the quality and integrity of the research. For instance, Newson *et al.* (2021) described, within the context of New South Wales, how the Australian Government’s drive for public health evidence to support its childhood obesity policies may have narrowed the research to specific topics and methodologies, thus preventing other less ‘desirable’ public health issues from receiving attention. Further, they identified that policy agencies, i.e. government-associated entities, frequently commission research on obesity to gain evidence to support pre-formed policy goals (Newson *et al.* 2021). Our review also revealed five instances where government influence was considered positive. For example, Gordon *et al.* (2018) describe a sexual health government-funded research project in which governments were constructively involved in priority setting and planning, allowing them to gain the key evidence they required. This greater integration between research and the use of evidence arguably resulted in improved population health outcomes. These examples illustrate how government involvement in research can be both beneficial and detrimental to the research and the population health outcomes, and highlight the difficulties in defining how the government should interact in public health research (Cairney and Oliver 2017).

### Government-commissioned research may be at risk

From this sample, only a small number of the included documents reported government influence on research funded via a commissioning arrangement. This may be unsurprising given that this type of funding arrangement constitutes only a minor proportion of total global public health research activities (CIC Collaborate 2017, OECD 2018a, 2018b). However, the limited availability of documents on this topic may also reflect the challenges that arise when commissioned research is undertaken. For instance, government influence may share similar reputational issues observed in commercially commissioned nutrition research (Cullerton *et al.* 2019) and alcohol industry research (McCambridge and Mitchell 2022). In these contexts, the researchers’ fear of experiencing ‘reputational harm’ or loss of future commissioned contracts may result in them avoiding reporting problematic interactions, leaving negative influence under-represented in the literature.

### Opposing perspectives on government influence

Our review revealed that the majority of the included documents described government influence as having a negative impact on the research overall. Importantly, these findings highlight that although it may be intuitive to assume that governments seek evidence that facilitates decisions to improve public health outcomes, other motivations beyond beneficial health outcomes may be present. For example, governments may manipulate public health research so that the findings align with their political requirements or may defer policy-related decisions, usually until after the next election (Barnish *et al*. 2018; Friel *et al.* 2023).

Building on this, due to the small sample of documents, it is unclear whether this negative perspective is widely shared amongst the public health community. Previous research investigating opinions on commissioned research suggests that a diversity of perspectives exists, ranging from wholly supportive to wholly against this form of research (Cullerton *et al.* 2019). These differences in perspective are captured by Warin and Moore (2021) in their report on how academic researchers and government employees frequently clashed in their views on how to conduct the research in ways that preserved academic rigour. The authors describe the multiple subtle ways that the government deviated from the research process, many of which went unnoticed by those involved. In a much more apparent act of influence, Storeng and Palmer (2019) describe how global health researchers disagreed with the funding entity when the funder attempted to block the publication of the research findings. Both of these examples demonstrate the ethical complexities around influence on the research process: one entity may consider the influence justifiable or acceptable, whereas another may consider the influence ‘undue’ or unethical (Halilem 2010; D’Este *et al.* 2018).

In sum, our findings confirm that government involvement in public health research can bring many potential benefits to populations, governments and researchers, but doing so can be challenging. Specifically, achieving a balance between research that brings positive population health outcomes whilst respecting the presence of political imperatives and personal career and credibility requirements is complex. Our review demonstrates this complexity; the included documents report multiple ways that governments can influence public health research, cover a range of public health topics and present a diversity of perspectives on its impact on research directions and outcomes.

### Strengths and limitations

To our knowledge, this is the first study to systematically review and summarize the available literature relating to government influence on the public health research process. To do this, we extensively searched multiple databases and grey literature, using broad, non-specific search terms to capture the diversity of literature related to ‘influence’. To facilitate the conceptualization and understanding of how government influence manifests in public health research, we categorized the modes of influence using a novel framework (‘Direct’, ‘Indirect’, and ‘Subtle’), informed by Vie’s examination of research integrity in commissioned research (Vie 2022). We chose to focus on the research process rather than the research agenda (the choices made about which specific topics and questions will be researched) to allow us to explore government influence in terms of research integrity and how influence manifests in public health research.

We also recognize that this scoping review has several limitations. First, few documents met the inclusion criteria, resulting in a small sample size. Consequently, this limits the generalizability of our findings and means we cannot estimate the relative frequency of government influence, the modes of influence, or the impact it has on outcomes. Second, our review included only items published in English, potentially narrowing the search to English-speaking countries. Third, to maintain the manageability of the volume of data, we limited the scope to government influence within the academic environment, thus potentially missing private and government research centers. We also recognize that our searches may not have captured literature concerning local government due to a lack of reporting or public availability of documents within this context. Fourth, most documents came from HICs (predominately the UK and Australia), potentially skewing the dataset. Fifth, preferential biases towards negative reporting and publishing may have led to an overrepresentation of negative influence. Conversely, positive modes of influence may be exaggerated, reflecting anxiety within the public health community that an individual’s criticism of the government might reduce their chances of future funding. Finally, we recognize that any author’s analysis of government influence will inevitably be partly subjective.

### Future directions

Due to the limited and heterogeneous documents included in this review, our findings do not necessarily represent all government influence on public health research. Instead, they provide a starting point from which to build a greater understanding of this issue. Additionally, our results revealed gaps in the existing literature. Namely, the extent of government influence globally or whether there are any associated factors, such as funding type, public health topic, or methodology. Also, this review uncovered the potential for government influence to manifest as clauses within government-funded contracts, although the literature regarding such contractual agreements and clauses is sparse. We, therefore, suggest that future research might be directed at establishing more firmly the extent of government influence, how this manifests within the public health research domain, any associated factors that increase the likelihood of influence, and the consequent impact on research outcomes.

The primary purpose of our review was to inform future guidance regarding government interactions with public health research. In recognition of this and acknowledging the predominantly negative outcomes reported on government influence, we recommend exploring ways to improve government funding arrangements to better protect academic research integrity. Such investigations could include creating more robust government-commissioned research contracts and the development of structured guidance on government-academic collaboration. In situations where government-commissioned public health research is problematic or not possible, alternative arrangements might be identified by exploring types of funding arrangements that are used in global locations where government commissioning is not typically available.

## CONCLUSIONS

This scoping review identified and summarized some of the ways government influence manifests in public health research. We revealed that governments can (and do) exert influence throughout the research process in diverse ways, some of which may be subtle or unintentional. Although government influence can be seen as positive, it was more frequently considered negative in our sample, subtly deviating the research process away from creating robust and credible evidence and potentially distorting the public health evidence base.

Importantly, our findings demonstrate that guidance is needed regarding promoting beneficial interaction between government and academic researchers to avoid modes of influence that are undue or problematic to academic research integrity. This insight may be particularly relevant to the public health community and those involved in government-academic research because it draws attention to the potential for governments to change the directions and processes of public health research. The limited number of included sources highlights that additional investigation is required to establish the extent and impact of this influence in more detail. However, we hope our review encourages more attentiveness to research integrity and promotes positive government interaction within government-academic public health research.

## Supplementary Material

daaf097_Supplementary_Data

## Data Availability

The data underlying this article are available in the article and in its online Supplementary material.
