# Supplementary material for: How governments influence public health research: a scoping review
Source: Health Promot Int. 2025 Jul 7;40(4):daaf097. doi: 10.1093/heapro/daaf097 (PMC12230708; doi:10.1093/heapro/daaf097)
Supplement: daaf097_Supplementary_Data [file daaf097_supplementary_data.zip › ScR manuscript-S1 final submittal.docx]

#### **Supplementary Material (S1): Population, Concept, and Context**

| Population, Concept, and Context Description **Population:** any public health research project that has been successfully (or unsuccessfully) influenced by the government (an individual or organisation employed by a government body or government-associated intermediary body).  **Concept:** any action (or inaction) that impacts (or has the potential to affect) the research direction or process and may be directly or indirectly exerted, intentional or unintentional, and occur at any stage of the research cycle.  **Context:** any local, regional, national, or international project conducted in academic institutions in any global location and setting.  [NB. Our review included government-commissioned public health research, which is defined as a project in which the research activities relate to a public health topic or issue and in which a government department, office, or government-related agency partly or wholly pays for the research.] |
| --- |
