# Supplementary material for: How governments influence public health research: a scoping review
Source: Health Promot Int. 2025 Jul 7;40(4):daaf097. doi: 10.1093/heapro/daaf097 (PMC12230708; doi:10.1093/heapro/daaf097)
Supplement: daaf097_Supplementary_Data [file daaf097_supplementary_data.zip › ScR manuscript-S2 final submittal.docx]

#### **Supplementary Material (S2): Eligibility Criteria**

| Eligibility criteria **Inclusion criteria:** Primary documents, including articles, studies, commentaries, editorials, reports, and papers in peer-reviewed journals or grey literature, reporting or describing any public health research within an academic institution where a government entity has attempted or succeeded in modifying the research direction or process at any stage in the research cycle. The influence may come from any government-associated individual or organisation. Documents in the English language. All publication dates. All dates when government influence occurred or was reported. All research methodologies, geographical locations or resource settings.  **Exclusion criteria:** Documents describing the concept or principles of influence on public health research or the potential for influence to occur. Documents that describe researcher misconduct or questionable research practices, the influence on policymaking and the policy cycle, or influence outside of the research process (e.g., the research agenda, government legislation, research impact, medical education, health service/implementation delivery or practice implications). All secondary literature, biomedical and pharmaceutical research, and literature where it is unclear whether it relates to public health. |
| --- |
