# Supplementary material for: How governments influence public health research: a scoping review
Source: Health Promot Int. 2025 Jul 7;40(4):daaf097. doi: 10.1093/heapro/daaf097 (PMC12230708; doi:10.1093/heapro/daaf097)
Supplement: daaf097_Supplementary_Data [file daaf097_supplementary_data.zip › ScR manuscript-S3 final submittal.docx]

#### **Supplementary Material (S3): Example Search Strategy**

| Search strategy for Embase (limited to title, abstract, keyword plus English language and Human) (‘population health’ OR ‘public health’ OR ‘public health’/exp)  AND  (academi* OR universit* OR research/exp)  AND  (government OR governments OR governmental OR ‘policy rel*’)  AND  (bias* OR censor* OR clearance OR conflict* OR corrupt* OR ethic* OR governance OR hinder* OR influen* OR integrity OR interfer* OR involv* OR manipulat* OR misconduct OR modif* OR silenc* OR suppress* OR tension* OR transparen* OR “vested interest*”) |
| --- |
