# Supplementary material for: How governments influence public health research: a scoping review
Source: Health Promot Int. 2025 Jul 7;40(4):daaf097. doi: 10.1093/heapro/daaf097 (PMC12230708; doi:10.1093/heapro/daaf097)
Supplement: daaf097_Supplementary_Data [file daaf097_supplementary_data.zip › ScR manuscript-S4 final submittal.docx]

#### **Supplementary Material (S4): Characteristics of the Project(s)**

| **Citation** | **Country of reported influence** | **Socio-economic status of the country(ies)** | **Project remit** | **Type of funding arrangement** | **Topic of research** | **Type of research** |
| --- | --- | --- | --- | --- | --- | --- |
| (The LSE GV314 Group, 2014) | UK | HIC | Multiple | Government-commissioned research | Multiple (including Public Health) | Policy and Programme Evaluation |
| (Gordon *et al.*, 2018) | Australia | HIC | Regional | Government-funded | Blood-borne viruses and sexually transmissible infections | Multiple |
| (Gornall, 2014) | UK | HIC | National | Government-commissioned | Alcohol | Data Modelling |
| (Haynes *et al.*, 2011) | Australia | HIC | Regional | Not disclosed | Public Health (including drugs, obesity, and tobacco) | Case Study |
| (Katikireddi *et al.*, 2014) | UK | HIC | National | Not disclosed | Alcohol | Econometric Modelling |
| (Kypri, 2015) | Australia | HIC | Regional | Researcher-initiated grant from a government entity | Alcohol | Programme Implementation |
| (McCrabb *et al.*, 2021) | Australia; Canada; UK; USA; other | HIC; MIC; LIC | Multiple | Not disclosed | Nutrition; physical activity; sexual health; smoking; substance use | Not disclosed |
| (Miller *et al.*, 2017) | Australia; Canada; UK; USA; other | HIC; MIC; LIC | Multiple | Multiple (including government-funded and government-commissioned) | Addiction science (including alcohol, tobacco, and drugs) | Multiple |
| (Newson *et al.*, 2021) | Australia | HIC | Regional | Multiple | Obesity | Multiple |
| (Ries and Kypri, 2018) | Australia | HIC | National | Government-funded | Health (including Public Health) | Research and Evaluation |
| (Sedley, 2016) | UK | HIC | National | Government-commissioned | Alcohol; nutrition; health services | Data Modelling |
| (Smith, 2014) | UK | HIC | National | Not disclosed | Health inequalities and health policy | Multiple |
| (Smith, 2010) | UK | HIC | National | Not disclosed | Health inequalities and health policy | Not disclosed |
| (Storeng and Palmer, 2019) | other | MIC; LIC | International | Government-commissioned | Sexual Health | Not disclosed |
| (Warin and Moore, 2021) | Australia | HIC | Regional | Government-funded | Obesity | Qualitative Ethnography |
| (Williamson *et al.*, 2019) | Australia | HIC | Multiple | Not disclosed | Health (including Public Health) | Not disclosed |
| (Yazahmeidi and Holman, 2007) | Australia | HIC | Multiple | Not disclosed | Public Health | Not disclosed |
