# Supplementary material for: How governments influence public health research: a scoping review
Source: Health Promot Int. 2025 Jul 7;40(4):daaf097. doi: 10.1093/heapro/daaf097 (PMC12230708; doi:10.1093/heapro/daaf097)
Supplement: daaf097_Supplementary_Data [file daaf097_supplementary_data.zip › ScR manuscript-S5 final submittal.docx]

#### **Supplementary Material (S5): Case Examples of Government Influence**

| **The nature of influence** | **Citation** | **The point of influence in the research process** | **Influencing action** |
| --- | --- | --- | --- |
| **Direct** | (Sedley, 2016) | Dissemination | **The government delayed disseminating findings to suit its political agenda:** During the UK's minimum unit pricing policy decision-making, the government specified the timing for publishing the research findings. |
| **Indirect** | (Kypri, 2015) | Inception | **The government entity inserted clauses into the funding contract:** The clauses in the government-academic funding contract enabled the government to terminate the research project without consultation, restrict/block the release of findings, and define dissemination methods. |
| **Subtle** | (Haynes *et al.*, 2011) | Throughout | **The researchers altered their behaviours to facilitate their involvement in policy-relevant research:** Government representatives engaged with academic researchers by asking for advice, guidance, and research evidence to assist in policy-making. During this engagement process, the government representatives communicated their preferred public health research outputs. In turn, the academic researchers (consciously or subconsciously) modified their funding applications and project choices to use research methodologies and present evidence in the government's desired forms. |
